# Community-based model for the delivery of antiretroviral therapy in Cambodia: a quasi-experimental study protocol

**DOI:** 10.1186/s12879-021-06414-y

**Published:** 2021-08-06

**Authors:** Sovannary Tuot, Alvin Kuo Jing Teo, Kiesha Prem, Pheak Chhoun, Chamroen Pall, Mengieng Ung, Penh Sun Ly, Masamine Jimba, Siyan Yi

**Affiliations:** 1 KHANA Center for Population Health Research, Phnom Penh, Cambodia; 2 Department of Community and Global Health, Graduate School of Medicine, The University of Tokyo, Tokyo, Japan; 3 Faculty of Social Sciences and Humanity, Royal University of Phnom Penh, Phnom Penh, Cambodia; 4 Saw Swee Hock School of Public Health, National University of Singapore and National University Health System, 12 Science Drive 2, #10-01, Singapore, 117549 Singapore; 5 Department of Infectious Disease Epidemiology, Faculty of Epidemiology and Population Health, London School of Hygiene and Tropical Medicine, London, UK; 6 Lee Kuan Yew Centre for Innovative Cities, Singapore University of Technology and Design, Singapore, Singapore; 7 National Center for HIV/AIDS, Dermatology and STD, Phnom Penh, Cambodia; 8 Center for Global Health Research, Touro University California, Vallejo, CA USA

**Keywords:** Cambodia, HIV, Community, Antiretroviral therapy, Service delivery, Implementation science

## Abstract

**Background:**

Multi-month dispensing (MMD) is the mainstay mechanism for clinically stable people living with HIV in Cambodia to refill antiretroviral therapy (ART) every 3-6 months. However, less frequent ART dispensing through the community-based ART delivery (CAD) model could further reduce the clients’ and health facilities’ burden. While community-based services have been recognized as an integral component of HIV response in Cambodia, their role and effectiveness in ART delivery have yet to be systematically assessed. This study aims to evaluate the CAD model’s effectiveness on the continuum of care and treatment outcomes for stable people living with HIV in Cambodia.

**Methods:**

We will conduct this quasi-experimental study in 20 ART clinics across the capital city and nine provinces between May 2021 and April 2023. Study sites were purposively selected based on the availability of implementing partners, the number of people living with HIV each clinic serves, and the accessibility of the clinics. In the intervention arm, approximately 2000 stable people living with HIV will receive ART and services from the CAD model. Another 2000 stable people living with HIV in the control arm will receive MMD—a standard care model for stable people living with HIV. The primary outcomes will be retention in care, viral load suppression, and adherence to ART. The secondary endpoints will include health providers’ work burden, the model’s cost-effectiveness, quality of life, mental health, social support, stigma, and discrimination. We will compare the outcome indicators within each arm at baseline, midline, and endline using descriptive and inferential statistics. We will evaluate the differences between the intervention and control arms using the difference-in-differences method. We will perform economic evaluations to determine if the intervention is cost-effective.

**Discussion:**

This study will build the evidence base for future implementation and scale-up of CAD model in Cambodia and other similar settings. Furthermore, it will strengthen engagements with community stakeholders and further improve community mobilization, a vital pillar of the Cambodian HIV response.

**Trial registration:**

ClinicalTrials.gov, NCT04766710. Registered 23 February 2021, Version 1.

**Supplementary Information:**

The online version contains supplementary material available at 10.1186/s12879-021-06414-y.

## Background

Cambodia has substantially reduced the prevalence of human immunodeficiency virus (HIV) in the general adult population aged 15–49 years from 1.7% in 1998 to 0.5% in 2019 [1, 2]. Cambodia is also one of the three countries in the Asia-Pacific region to have achieved the UNAIDS 90–90-90 targets. More than 90% of people living with HIV receiving antiretroviral therapy (ART), and 80% had viral suppression [3]. Despite the successes, there are persistent concerns regarding attrition from HIV testing and linkages to care and the retention in care, especially among members of the hidden populations in Cambodia [4]. A recent systematic review and meta-analysis of studies in low- and middle-income countries reported that groups with vulnerable socioeconomic and clinical status and social support were at increased risk of loss to follow-up from ART programs [5]. From the health systems perspective, care providence at a higher level was associated with a higher risk of attrition from the treatment cascade [5]. Therefore, through decentralization of care and community involvement, differentiated ART delivery models that are contextualized could improve care retention and sustain treatment outcomes [6, 7].

In Cambodia, ART is primarily administered at 68 public ART clinics across 25 provinces [8]. Clinics operated by several non-governmental organizations (NGOs) also complement the ART services. However, regular clinic runs and ARV refills posed a heavy resource burden on people living with HIV to remain optimally engaged in care [9]. Besides, it also contributed significant workload on the health facilities and service providers to meet the demands of both stable people living with HIV and unstable people living with HIV, who required more frequent follow-ups [10]. Hence, a differentiated care model would enable health providers to invest more time on unstable and complicated cases, thus improving overall service quality [10]. An appointment-spacing and multi-month dispensing (MMD) model has been introduced to all ART clinics to increase service efficiency and decrease congestion by reducing clinical visits and refill appointments for stable people living with HIV [11]. However, concerns regarding sub-optimal storage of antiretrovirals (ARVs), poor adherence, and missed appointments have emerged [12].

Community-based service delivery plays an integral part in the HIV response in many countries, including Cambodia [1, 13–17]. A study conducted among people living with HIV in Cambodia expressed the need for community-based interventions to reduce stigma and discrimination among the general public and support people living with HIV to address these stressful situations [18]. In 2016, the World Health Organization (WHO) recommended that stable people living with HIV can safely reduce the frequency of clinic visits by potentially receiving ART in community settings [13]. Compared to hospital-based settings, decentralizing HIV treatment delivery and task shifting to include community health workers to dispense ART was reported to improve retention across the HIV care cascade [19]. Community-based ART delivery (CAD) models implemented outside Cambodia have reduced burdens for people living with HIV and the health systems, increased retention in care, and lowered overall service provider costs [20–24].

The two promising differentiated care models (i.e., MMD and CAD) have yet to be fully implemented and evaluated in Cambodia. Hence, little is understood on the operationality and effectiveness of the models in improving the care of people living with HIV. The primary aim of this study is to implement and evaluate the effectiveness of CAD compared to MMD on the care continuum and treatment outcomes for stable people living with HIV. We will also determine the acceptability, feasibility, barriers, and facilitators to implementing the CAD model in Cambodia. We hypothesize that a significantly higher proportion of participants in the intervention arm will report viral load suppression, retention in care, good ART adherence, improved quality of life and mental health, a better sense of social support, and lower stigma and discrimination. We also hypothesize that the CAD model will be more cost-effective compared to MMD (control arm). A significantly higher proportion of health care providers at the ART clinics in the intervention arm is also postulated to report reduced workload and lower burnout scores due to the implementation of CAD.

## Methods

### Design

We will conduct a quasi-experimental study [25, 26] consisting of two arms—intervention and control for 24 months. Approximately 2000 stable people living with HIV in the intervention arm will receive services from the CAD model. Another 2000 people living with HIV in the control arm will receive MMD—a default care model for stable people living with HIV to refill ART every 3-6 months with a routine 6-monthly clinical review. We will adopt an effectiveness-implementation hybrid design type II [27], where a mixed-methods approach will run in parallel to evaluate the effectiveness (quantitative) and implementation strategy (qualitative).

### Settings

This study will include 10 ART clinics in Phnom Penh capital city and four provinces (Kampong Thom, Kampot, Koh Kong, and Takeo) for the intervention arm (Table 1). We will include 10 other ART clinics in Phnom Penh and five provinces (Kampong Cham, Pailin, Preah Sihanouk, Siem Reap, and Prey Veng) for the MMD control arm (Table 1). These sites have been purposively selected based on (i) the availability of implementing partners (Cambodian People Living with HIV Network, ARV User Association, and Partner in Compassion), (ii) the number of eligible people living with HIV for enrollment in the intervention and control arms, and (iv) advice from the national HIV program on the accessibility of the clinics.

**Table 1:** Study sites for the quasi-experimental study to evaluate the CAD model

Intervention sites			Control sites		
Province	ART clinic	Setting	Province	ART clinic	Setting
Kampot	Kampot Hospital	Urban	Kampong Cham	Kampong Cham Hospital	Urban
Kampot	Kampong Trach Hospital	Rural	Kampong Cham	Cheung Prey Hospital	Rural
Koh Kong	Smach Meanchey Hospital	Urban	Pailin	Pailin Hospital	Urban
Koh Kong	Sre Ambil Hospital	Rural	Preah Sihanouk	Preach Sihanouk Hospital	Urban
Phnom Penh	Mean Chey Hospital	Urban	Phnom Penh	Chhouk Sar I Clinic	Urban
Phnom Penh	Pochintong Hospital	Urban	Phnom Penh	Sam Dach Ov Hospital	Urban
Kampong Thom	Kampong Thom Hospital	Urban	Siem Reap	Siem Reap Hospital	Urban
Kampong Thom	Baray Santok Hospital	Rural	Siem Reap	Soth Nikum Hospital	Rural
Takeo	Takeo Hospital	Urban	Prey Veng	Prey Veng Hospital	Urban
Takeo	Kirivong Hospital	Rural	Prey Veng	Neak Loeung Hospital	Rural

*ART* antiretroviral therapy, *CAD* community ART delivery

### Participants

We define stable people living with HIV as individuals who (i) are ≥15 years, (ii) have received first-line ART for at least 1 year, (iii) did not report ART-related adverse reactions or drug interactions requiring regular monitoring; (iv) do not have tuberculosis (presumptive/confirmed), other opportunistic infections, and are not taking any prophylactic treatment; (v) are not pregnant or breastfeeding (for women); (vi) have a good understanding of lifelong treatment and adherence to medication; and (vii) have achieved treatment success—two consecutive undetectable viral load and/or CD4 counts above 200 cells/mm^3^ [11, 19, 28]. We will conduct in-depth interviews and focus group discussions with people living with HIV, representatives of the communities, government officials at different levels (community, health centers, operational districts, provincial health departments, and national programs), local and international NGOs, donors and other developmental partners in Cambodia to explore the acceptability, barriers, and facilitators to implementing the CAD model.

### Sample size calculation

A target sample size of approximately 2000 stable people living with HIV is required to provide at least 80% power to detect a 10% relative difference retention in care or maintenance of viral suppression. The sample size calculation was based on 10% attrition and conservative estimates of retention in care or viral-suppression maintenance: 50 and 55% maintenance of viral suppression in the MMD (control arm) and CAD (intervention arm), respectively. Findings from a recent randomized controlled trial in high- and medium-HIV-prevalence settings of people living with HIV with detectable viral load showed that CAD increased viral suppression compared to the control group (74% vs. 63%, RR 1.18, 95% CI 1.07–1.29). Under similar scenarios, our proposed study’s power with 2000 participants in each arm will be at least 90%.

### Recruitment

We will develop a list of participating ART clinics in consultation with the Database Management Unit of the National Center for HIV/AIDS, Dermatology, and STD (NCHADS) and the other implementing partners. Potential people living with HIV will be jointly identified by the study team and clinicians on-site based on the eligibility criteria. We will seek consent from eligible people living with HIV and recruit them into the study. To measure the impact of CAD on the work burden of healthcare providers, we will recruit all staff working for the HIV program at the intervention and control sites—nurses, counselors, laboratory technicians, pharmacists, and clinicians. For the qualitative study, we will purposively recruit interviewees at the national level and intervention sites for diverse views. Schedule of enrolment, intervention, and assessments is shown SPIRIT Figure (Supplementary Material 1).

### Intervention: CAD model

**Fig. 1:**
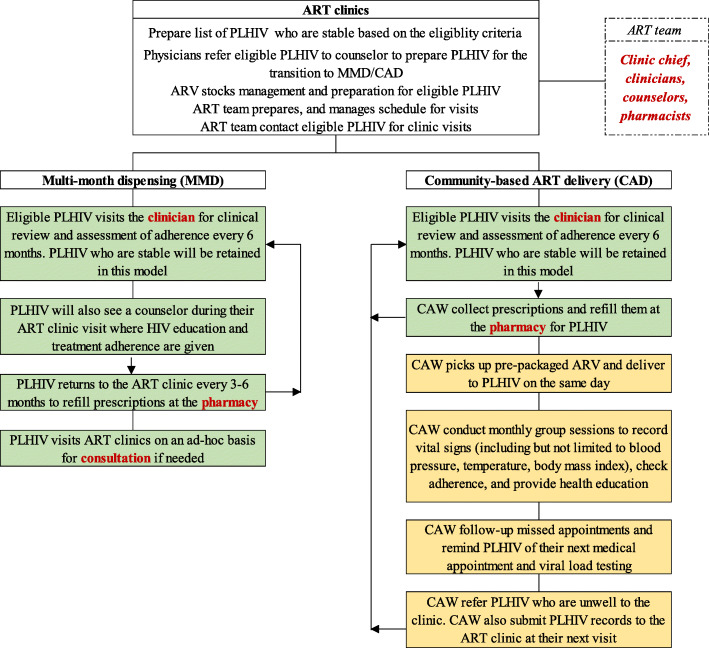
Study flowchart in the intervention (community ART delivery) and control arms (multi-month dispensing model). Notes: The department/profiles in red represent the workforce required at the ART clinics to facilitate and implement MMD and CAD. Boxes in green represent activities that will occur in the ART clinics. Boxes in yellow represent activities that will occur in the community

People living with HIV in the intervention arm will receive antiretrovirals (ARVs) from the community action workers (CAWs) through the CAD model. We will recruit people living with HIV in the community to be CAWs. Training will be provided by the ART clinics and implementing partners on ART dispensing, drug storage, vital signs assessment and documentation, HIV education and counseling, ART adherence and measurement, referral systems, and aspects such as mental health, stigma and discrimination, and sexual and reproductive health. CAWs will obtain pre-packaged ARVs once a month from the attached ART clinic and dispense them to people living with HIV in the community. CAWs will conduct group follow-up sessions monthly to record vital signs, monitor adherence, and provide health education and counseling. Those who are unwell will be referred to the ART clinic. CAWs will also submit the relevant records to the ART clinic at their next visit. Trained clinicians will conduct ARV regimen and HIV clinical management in the ART clinics. People living with HIV in the intervention arm will visit the ART clinics for routine clinical review every 6 months and on an ad-hoc basis if medical attention is required. The study flowchart is presented in Fig. 1.

### Control: MMD model

People living with HIV in the control arm will visit ART clinics and collect their ARVs from the facility-based service providers through the MMD model, the standard care for stable people living with HIV. People living with HIV will refill their prescriptions at the ART clinic every 3-6 months. The exact frequency might differ between individuals, decided at the discretion of individual ART clinics. People living with HIV will visit the ART clinic on an ad-hoc basis for consultation if needed. They will otherwise undergo routine clinical review every 6 months. Clinical management of people living with HIV in the intervention and control group will be done by trained personnel at the ART clinic following the national guideline [28].

### Outcome measures

**Fig. 2:**
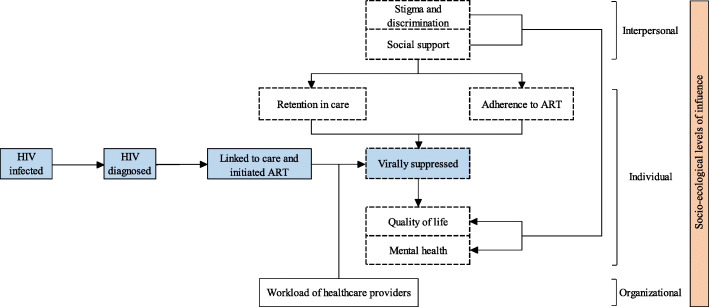
Conceptual framework reflecting the outcome measures and their effects on the continuum of care. Note: The framework outlines the relationship between the outcome measures (boxed in dotted line) and the HIV continuum of care (blue). The right-most panel (orange) represents the outcome measures in the context of the socioecological levels of influence

We present the conceptual framework reflecting the outcome measures and their effects on the continuum of care in Fig. 2. Our primary outcomes will include (i) viral load suppression, (ii) retention in care, and (iii) adherence to ART. Viral load suppression is defined as at least 90% of the study participants in the intervention arm will have a viral load of < 1000 RNA copies/mL at the end of the intervention period. We define care retention as the proportion of people living with HIV who will remain in HIV care and treatment 12 and 24 months after study commencement. Treatment adherence will be self-reported, and we will present the proportion of people living with HIV with good adherence to ART based on standard cut-offs.

We will also assess secondary outcomes, including (i) the impact of CAD on the work burden of healthcare providers at the ART sites, (ii) cost-effectiveness of this model and its impact on (iii) the quality of life, (iv) mental health, (v) social support, and (vi) stigma and discrimination faced by people living with HIV in the community. We will report the time and volume of stable people living with HIV seen at the ART sites as a measure of the workload of healthcare providers in HIV care delivery. We will present quality of life, mental health, social support, stigma and discrimination, and workload (burnout) as scores measured using validated tools described in the section below. We will perform an economic evaluation to determine if the intervention is cost-effective. We will also determine the acceptability, feasibility, barriers, and facilitators to implementing the CAD model in Cambodia using the qualitative method.

### Data collection

**Fig. 3:**
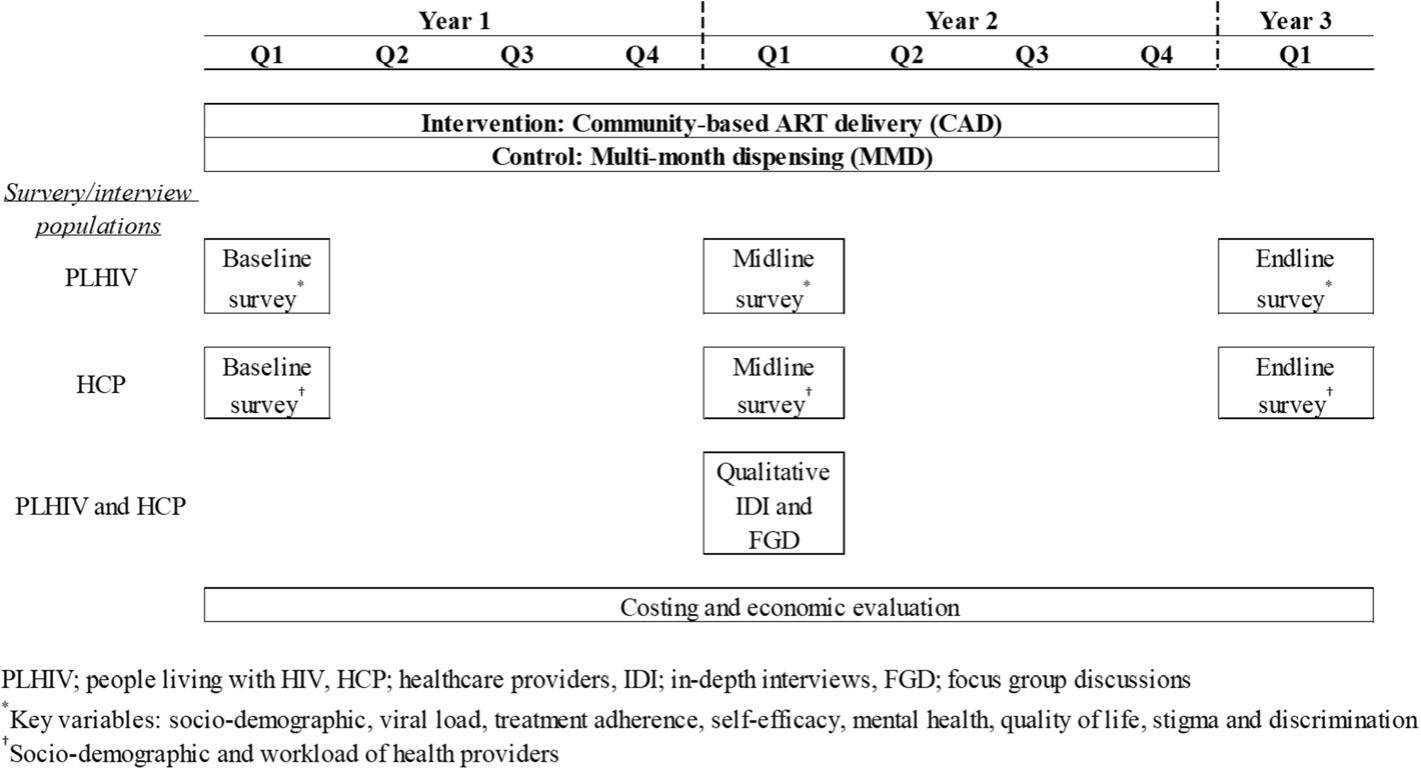
Timelines and data collection activities for the quasi-experimental study to evaluate the community ART model

All people living with HIV will be interviewed face-to-face by trained data collection teams using structured questionnaires. We will interview all participants in the intervention and control arms at baseline (0 months), midline (at 12 months of the intervention), and endline (at 24 months of the intervention). We will collect qualitative data in private locations using semi-structured interview guides. The data collection timelines for quantitative surveys and qualitative in-depth interviews and focus group discussions are illustrated in Fig. 3.

### Variables and measurements

We will collect information on sociodemographic (age, sex at birth and gender, membership of key population groups, income, residence, education, family, and dependency) and HIV treatment history and health status (duration of taking ART, transportation mode, relationship, and satisfaction with health care workers). We will adapt questionnaires previously implemented by NCHADS to measure medication adherence and retention in care and treatment [28, 29]. Viral load will be obtained from medical records at the attached ART clinics.

We will measure depressive symptoms using the Center for Epidemiologic Studies Depression Scale (CES-D) [30], quality of life using the WHO Quality of Life BREF (WHOQOL-BREF) for people living with HIV and EQ-5D-5L [31–33] and stigma and discrimination using the People Living with HIV Stigma Index [18]. We will also measure self-efficacy and levels of social support using the Perceived Self-Efficacy for Receiving Antiretroviral Therapy Scale (PSEARTS) [34–36] and Berlin Social Support Scale (BSSS) [35, 37, 38], respectively. For the economic evaluation, we will collect direct and indirect costs for follow-up care and ARV refills. We will assess healthcare providers’ workload at ART clinics using two different measures. First, we will evaluate health providers' workload and burnout using the NASA Task Load Index [39] and a single-item measure of burnout adapted from the Physician Worklife Study [40–43], respectively. Second, we will measure the number of people living with HIV (both stable and unstable) served per day and week and the time spent per people living with HIV at the different service points in the clinic. For the qualitative assessment, we will explore the perception of people living with HIV, HIV service providers, and other key stakeholders on the advantages and disadvantages, acceptability, feasibility, and sustainability of CAD in the HIV continuum of care.

### Data management

We will collect and manage data using REDCap [44, 45]. Data coding, quality control, and data entry will be done. We will enter all data into the database within 1 week of collection. Subsequently, we will export the database into Microsoft Excel (Microsoft Corp., Redmond, Washington, USA) to check for consistency periodically. All questionnaires will be checked for errors by the team leaders, and necessary corrections will be made before data entry and analyses.

### Data analyses

We will compare the characteristics of the participants in the intervention and control arms using descriptive statistics. In the presence of significant differences, we will adjust for them in the subsequent between-group (i.e., intervention vs. control) analyses. Key outcome indicators will be compared using inferential statistics such as Chi-square tests for categorical variables and Student’s *t*-test for continuous variables. To evaluate the effectiveness of the CAD model intervention, we will compare the outcome indicators at baseline, midline, and endline within and between the intervention and control arm using the difference-in-differences method [46]. We will apply multivariable regression analyses using mixed models, logistic and Cox regression, considering repeated measurements design and the nature of the response variables. Statistical analyses will be performed in STATA (Stata Corp LP, Texas, United States of America) and R (R Foundation for Statistical Computing, Vienna, Austria). For qualitative data, we will transcribe audio recordings verbatim, and the transcripts will be coded using NVIVO (QSR International). Content analyses will be performed to identify emerging categories, themes, and common and divergent patterns pertinent to the study’s objectives.

### Ethical consideration

We will inform all participants clearly about the study’s objectives and the participation risks and benefits before enrolling them in the study (Supplementary Material 2). If a participant cannot read or write, the CAWs or data collectors will read the information sheet to the participant during the consent-taking process. Participants will be able to withdraw from or discontinue the study at any time. All participant records—written, recorded, and transcribed data—will be stored securely. We will assign coded identifiers to participant names (with a master list stored separately). Participants will receive a token equivalent to US$5 for their time and transportation compensation. It is anticipated that the study results will benefit people living with HIV and their communities, and the harm to the participants will be minimal.

## Discussion

As a country with a majority of people living with HIV being aware of their status, undergoing treatment, and achieving viral suppression [3], it is essential to ensure that people living with HIV remain in care and virally suppressed. Hence, a decentralized and differentiated model catering to stable people living with HIV ought to be prioritized. This study seeks to operationalize CAD and examine its effects on the care continuum and treatment outcomes for stable people living with HIV in Cambodia, hinging on the successes of community-based service delivery mechanisms [21–24]. We hypothesize that the CAD model will lead to better viral suppression, ART adherence, care retention, and improvement in the quality of life, mental health, and social support of people living with HIV. We also theorize that the community-based model will reduce healthcare providers’ workload at the ART clinics and stigma and discrimination among people living with HIV. The model is also postulated to be cost-effective, measured in terms of dollar spent per additional quality-adjusted life-years gained.

A limitation of our approach is the lack of randomization. Hence, we cannot definitively account for unmeasured and residual confounders. Cambodia’s Ministry of Health operates the ART clinics under support from the Global Fund to Fight AIDS, Tuberculosis, and Malaria, the country’s largest funder of HIV programs. The Global Fund works with the NCHADS and other sub-sub implementers to support HIV treatment, care, and support across all 68 ART sites in the country. The programs include the provision of HIV counseling and education session to people living with HIV, facilitation of access to ART services, and the provision of living support to people living with HIV. All 20 ART clinics selected for this study receive a similar level of support from the Global Fund-supported programs. Therefore, these activities will be carried out in the backdrop of this evaluation.

Potential contamination confers by the HIV program supported by the Global Fund could bias the intervention effect towards null and may lead to a type 2 error. However, the contamination is likely non-differential between the intervention and control sites. Study data could also be subjected to information bias as data will be collected by interviewers, and several outcomes will be based on respondents’ recall and self-report. Data collectors in both arms will be trained in a standardized manner to minimize information bias. We will also ensure that the participants, data collectors, and implementation teams will be blinded to the study’s main hypotheses.

This project complements the national HIV program’s efforts to meet and sustain targets to end HIV and AIDS in Cambodia by ensuring that people living with HIV stay on treatment and their viral load is suppressed. Through community mobilization, the differentiated ART delivery model could also allow the apportionment of resources to intensify case finding and ensure that more people living with HIV would transition to a stable stage. Lessons learned from this study will be documented and disseminated to different stakeholders. Upon completion of this study, the findings will inform the implementation and enable scale-up of CAD in other localities in Cambodia and other similar settings.

## Supplementary Information

**Additional file 1.** SPIRIT Figure Schedule of enrolment, intervention, and assessments.

**Additional file 2.**

## Data Availability

Not applicable.

## Abbreviations

ART: Antiretroviral therapy
ARV: Antiretroviral
BSSS: Berlin Social Support Scale
CAD: Community-based ART delivery
CAW: Community action workers
CES-D: Center for Epidemiologic Studies Depression Scale
HIV: Human immunodeficiency virus
MMD: Multi-month dispensing
NCHADS: National Center for HIV/AIDS, Dermatology, and STD
NGO: Non-governmental organizations
PSEARTS: Perceived Self-Efficacy for Receiving Antiretroviral Therapy Scale
WHO: World Health Organization
WHOQOL-BREF: WHO Quality of Life BREF
