# Supplementary material for: Community-based model for the delivery of antiretroviral therapy in Cambodia: a quasi-experimental study protocol
Source: BMC Infect Dis. 2021 Aug 6;21:763. doi: 10.1186/s12879-021-06414-y (PMC8344198; doi:10.1186/s12879-021-06414-y)
Supplement: Supplementary file 1 — Additional file 1. SPIRIT Figure Schedule of enrolment, intervention, and assessments. [file 12879_2021_6414_MOESM1_ESM.docx]

**Supplementary Material 1**

**SPIRIT Figure Schedule of enrolment, intervention, and assessments**

|  | **Study period** | | | | | | | | | | | | | |
| --- | --- | --- | --- | --- | --- | --- | --- | --- | --- | --- | --- | --- | --- | --- |
| **Timepoint** | **Enrolment** | | **Allocation** | | **Post-allocation** | | | | | | | | **Close-out** | |
|  | **‘20** | **‘20** | **‘21** | **‘21** | **‘21** | **‘21** | **‘22** | **‘22** | **‘22** | **‘22** | **‘23** | **‘23** | **‘23** | **‘23** |
|  | **Q3** | **Q4** | **Q1** | **Q2** | **Q3** | **Q4** | **Q1** | **Q2** | **Q3** | **Q4** | **Q1** | **Q2** | **Q3** | **Q4** |
| **Enrolment**  **(individual level)** |  |  |  |  |  |  |  |  |  |  |  |  |  |  |
| Assessment of eligibility (clinical screening) | X | X |  |  |  |  |  |  |  |  |  |  |  |  |
| Inform consent |  |  | X | X |  |  |  |  |  |  |  |  |  |  |
| Allocation of intervention |  |  |  | X |  |  |  |  |  |  |  |  |  |  |
| **Interventions** |  |  |  |  |  |  |  |  |  |  |  |  |  |  |
| Community-based ART delivery model |  |  |  |  | X | X | X | X | X | X | X | X |  |  |
| Multi-month dosing model |  |  |  |  | X | X | X | X | X | X | X | X |  |  |
| **Assessments**  **(primary outcomes)** |  |  |  |  |  |  |  |  |  |  |  |  |  |  |
| Viral load suppression |  |  |  | X |  |  |  |  | X |  |  |  | X |  |
| Retention in care |  |  |  | X |  |  |  |  | X |  |  |  | X |  |
| Adherence to ART |  |  |  | X |  |  |  |  | X |  |  |  | X |  |
| **Assessments**  **(secondary outcomes)** |  |  |  |  |  |  |  |  |  |  |  |  |  |  |
| Work burden of healthcare providers |  |  |  | X |  |  |  |  |  |  |  |  | X |  |
| Costs data |  |  |  | X |  |  |  |  |  |  |  |  | X |  |
| Stigma and discrimination |  |  |  | X |  |  |  |  | X |  |  |  | X |  |
| Social support |  |  |  | X |  |  |  |  | X |  |  |  | X |  |
| Quality of life |  |  |  | X |  |  |  |  | X |  |  |  | X |  |
| Mental health |  |  |  | X |  |  |  |  | X |  |  |  | X |  |
| **Assessment**  **(Qualitative IDI and FGD)** |  |  |  |  |  |  |  |  | X |  |  |  |  |  |

ART; antiretroviral therapy, IDI; in-depth interviews, FGD; focus group discussions
