# Supplementary material for: Community-based model for the delivery of antiretroviral therapy in Cambodia: a quasi-experimental study protocol
Source: BMC Infect Dis. 2021 Aug 6;21:763. doi: 10.1186/s12879-021-06414-y (PMC8344198; doi:10.1186/s12879-021-06414-y)
Supplement: Supplementary file 2 — Additional file 2. [file 12879_2021_6414_MOESM2_ESM.docx]

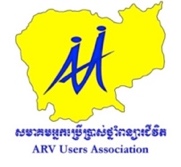

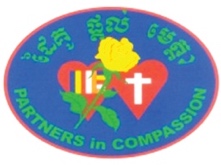

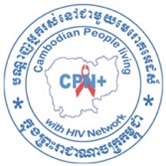

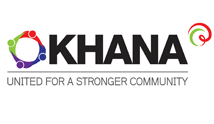

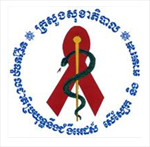


**Community-based model for the delivery of antiretroviral therapy in Cambodia: a quasi-experimental study protocol**

Information Sheet and Consent Form for Project Participants

ARV Code: …………………….

Name of ART Clinic: ……………………. Province: …………………….

Background/purpose of the project

You are invited to participate in the CAD project. Before you can participate in this project, the project must be explained to you, and you must be given a chance to ask questions. KHANA and its implementers, namely AUA, PC, and CPN+, in collaboration with the National Center for HIV/AIDS, Dermatology and STD (NCHADS) agreed to demonstrate a project in your community. The project will focus on maintaining viral suppression and retention in care, maintaining adherence to ART, reducing the work burden of healthcare workers at the ART sites, and improving the quality of life, social support, and mental health of people living with HIV.

Please read carefully the information provided here. If you agree to participate, please sign the consent form. You will be given a copy of this document to take home with you. This project aims to evaluate the effectiveness of the CAD model on the care continuum and treatment outcomes for stable people living with HIV.

Study procedures

The project duration is 36 months, with 24 months of intervention.

**Project benefits**

Our team will register you into our project with your permission, and your medical appointment will be rescheduled to four to six months, but you can still visit your doctor anytime if you feel unwell. From now on, you will be receiving your pre-packaged dispensing ARV, additional educations, counselling, psychological supports, routine health checking from your ART group leader at a pick-up point close to your home.

Participant’s right

When you sign this consent form in the end, it means that you have read this form and authorize the use and sharing of your protected health information, as explained below. Your secure health information will be kept confidential, and your identity will not be revealed in any publication or presentation of the results of this project.

Your participation in this pilot project is entirely voluntary. It is your choice not to participate in any or all parts of this program. Choosing not to participate will not affect your eligibility to access any public health services or future programs. If you decided to participate, you consent to your personal information, including test results, shared with the government health system and KHANA. Aggregated, non-identifying data from this project may be published or shared in the form of public reports, articles, or any other media.

Questions

If you have any questions about any aspect of this project, please refer your query to the Principal Investigator, Dr. Siyan Yi by Email: [siyan@doctor.com](mailto:siyan@doctor.com) or Project Coordinator, Mr. Tuot Sovannary by Email: [tsovannary@khana.org.kh](mailto:tsovannary@khana.org.kh) or telephone: 012 836 926.

**Project requirements**

Before joining in this project, we need to know something from you as below:

| **Item / Participants** | **Intervention Group** | | **Control Group** | |
| --- | --- | --- | --- | --- |
|  | **Yes** | **No** | **Yes** | **No** |
| I confirm that I don’t have a plan to move out from your current location for 24 months later |  |  |  |  |
| I consent to CAW (ART group leader) take packaged ARV from the clinic on behalf of me |  |  |  |  |
| I consent to CAW (Group leader) home visits and SMS or phone calls |  |  |  |  |
| I will join the group education sessions and other project activities if applicable |  |  |  |  |
| I will join saving groups if possible |  |  |  |  |
| I consent to participate in any survey conducted by the project |  |  |  |  |
| I consent to the project to collect and use personal health information |  |  |  |  |
| I consent to the project to use my photo or video if any requirement |  |  |  |  |

Consent to participate in the project

At this moment, I confirm that I fully understand the project's objectives and consent to participate in this project, as in my declaration above and my certified thumbprint below.

_________________________________________ ________________________

Signature/thumbprint of participant Date

_________________________________________ ________________________

Signature/thumbprint of community ART workers Date
